# Arthroscopy of septic carpitis in donkeys (*Equus asinus*)

**Published:** 2014-11-28

**Authors:** A.H. Elkasapy, A.I. AbdelGalil, A.M. Al-Akraa, I.M. Ibrahim, S.F. Ismail

**Affiliations:** 1 *Department of Surgery, Faculty of Veterinary Medicine, Benha University, Egypt*; 2 *Department of Surgery, Anesthesiology and Radiology, Faculty of Veterinary Medicine, Cairo University, Egypt*

**Keywords:** Arthroscopy, Diagnosis, Donkeys, Septic arthritis

## Abstract

Experimental septic arthritis was induced in the radiocarpal joint of 18 donkeys by intra-articular injection of *Staphylococcus aureus* (3-4X10^6^ CFU). The inoculated animals were divided into three groups (6 donkeys in each group). The arthroscopic examination was carried out before induction of septic carpitis and 3 days (group I), 14 days (group II), and 28 days (group III) after induction of infection. The arthroscopic examination of group I revealed hyperemia of synovial membrane and hypertrophied villi. In group II, severe hyperemia of synovial membrane, hypertrophied villi, pannus in the joint cavity and beginning of articular cartilage erosion were found. In group III, severe hyperemia of synovial membrane, hypertrophied villi and more prominent articular cartilage erosion were present.

## Introduction

Septic arthritis can be caused by hematogenous infection, traumatic injury, or itrogenic infection (Honnas *et al.*, 1992). In adult horses, direct trauma is the most common cause of infectious arthritis where tissue destruction and cellulitis can lead to an open joint and subsequently cause infectious arthritis (McIlwraith, 1987). The most common microorganism isolated from donkeys suffering from septic carpitis was *Staphylococcus aureus* (El-Maghraby and Al-Bawa’neh, 2002).

Arthroscopy provides a far more thorough examination of the carpal joints than arthrotomy by effectively moving the light source and thus enabling the examiners a closer inspection into the joint. Complete examination of the joint with the use of an arthroscope cannot be emulated by any arthrotomy incisions (McIlwraith *et al.*, 2005).

Magda *et al*. (2005) stated that the arthroscopic examination of the radiocarpal joint of the donkey from the lateral portal revealed that the medial joint angle formed by the distal surface of the radius and the proximal surface of the radial carpal bone was first seen. They also added that arthroscopic examination of the radiocarpal joint from the medial portal revealed that the first area seen was the lateral joint angle formed by the proximal articular surface of the intermediate carpal bone and the distal articular surface of the radius.

Arthroscopy is considered the most valid method for cartilage evaluation, but in case of small cartilage lesions there is a risk of overestimation of the defect size. The advantage of arthroscopy compared to the other diagnostic modalities is the possibility of immediate treatment of the identified joint problem (Niemeyer *et al.*, 2011; Spahn *et al.*, 2011).

Adequate arthroscopic examination of the intercarpal or radiocarpal joint is possible through a single dorsal arthroscopic portal for each joint. Using two dorsal separate portals improves visualization by increasing freedom of movement because of reduced soft tissue tension around the arthroscope and a reduced tendency to slip out of the joint when examining areas close to the arthroscopic portal (McIlwraith *et al.*, 2005).

Acute synovial infection is characterized arthroscopically by severe synovitis. Established infection frequently results in the production of an intrasynovial fibrinocellular conglomerate (pannus). This may cover the foreign material and devitalised tissues, act as a nidus for bacterial multiplication and is rich in inflammatory cells, degradative enzymes, and radicals.

It is also a barrier to synovial membrane diffusion, thus compromising further intrasynovial nutrition and limiting the access for circulating antimicrobial drugs. The quantum and the nature of the pannus appears to be dependent on the type and number of infecting organisms (Wright, 2002).

Arthroscopic examination of septic arthritis of the tarsocrural joint in horses revealed erosions and irregularity of articular surface, fissure, articular degeneration and hypertrophied villi (AbdEl-Glil, 2012).

Borg and Carmalt (2013) concluded that joint infection rate in the horse population that had elective arthroscopy without antimicrobial prophylaxis compared favorably with other reports citing 0.9% sepsis in horses after arthroscopy.

This study was aimed to describe the arthroscopic anatomy of the radiocarpal joint in donkeys, and to shed light on the arthroscopic assessment of induced septic arthritis *(Staph. aureus)* of the radiocarpal joint in donkeys.

## Materials and Methods

The present study was experimentally induced on 32 healthy, adult donkeys of both sexes. Fourteen animals were used for serial propagation of the *Staph. aureus* until the infective inoculums were obtained. The remaining 18 donkeys were divided into three groups; each group consisting of 6 animals. In these animals, induction of septic carpitis in the radiocarpal joints of donkeys by *Staph. aureus* was induced. After the induction of septic carpitis, arthroscopic examinations were performed.

These animals were divided into three groups. The examination of the three infected groups was done 3, 14 and 28 days after the induction of septic carpitis of the radiocarpal joint. The ethical committee in the Faculty of Veterinary Medicine, Benha University approved this work.

### Induction of septic arthritis (septic carpitis)

The induction of septic arthritis in donkeys was performed using a viable *Staph. aureus* colony (standard colony). *Staph. aureus* used for induction of septic arthritis in this study was isolated from a donkey suffering from septic arthritis. A sample from the infected synovial fluid of the carpal joint was aspirated, inoculated into nutrient broth and incubated for 24 hours at 37°C. Each radiocarpal joint was inoculated with 3-4X10^6^ CFU of viable *Staph. aureus*.

### Diagnostic arthroscopy of the carpal joints

### Anesthesia of donkeys for arthroscopic surgery

Arthroscopic examination was performed on donkeys under the effect of xylazine HCl (1mg/kg) as the tranquilizer and thiopental sodium (5% solution intravenously with a dose of 6-8 mg/kg body weight).

### Preparation of the carpus for arthroscopy

The carpus was clipped circumferentially from the proximal metacarpus to the distal radius, cleaned and draped with sterile towel immediately prior to surgery. The donkeys were placed in dorsal recumbency and hooves were suspended on bar for ease of control of the degree of flexion of the joint.

### Arthroscopic procedures

The arthroscope was inserted in the radiocarpal joint while the joint was 20 degree flexed. Two arthroscopic portals (lateral and medial) were used for each joint. The lateral portal was located half way between the tendons of the extensor carpi radialis and the common digital extensor tendon. The medial portal was located at about 1 cm medial to the tendon of extensor carpi radialis.

Distension of the joint was obtained with 15-20 ml of 0.9 normal saline using an 18 gauge needle inserted at the proposed site for insertion of the arthroscope mentioned above. The arthroscopic sleeve was inserted through a 1 cm skin incision at the site of needle insertion. With the aid of a sharp obturator, the sleeve was advanced into the joint until the fluid began to escape from the cannula. The sharp obturator was replaced with a 25 & 4 mm, forward oblique viewing arthroscope. An ingress fluid line and fiber optic light cable were attached to the arthroscope. The joints were viewed with a video camera and a monitor. The joints were irrigated throughout the procedure with normal saline delivered from an infusion set.

## Results

The serial propagation of *Staph. aureus* in donkey’s carpal joints revealed that the infective dose of septic carpitis was 3-4X10^6^ CFU.

### Arthroscopic examination of radiocarpal joint

### Arthroscopic examination of the radiocarpal joint from the lateral portal

**Fig. 1:**
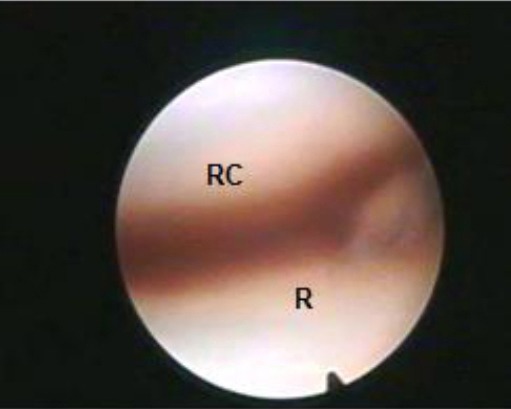
Arthroscopic view of the radiocarpal joint showing the medial joint angle. (R): Radius; (RC): Radial carpal bone.

Arthroscopic examination of the radiocarpal joint from the lateral portal revealed that the medial joint angle formed by the distal surface of the radius and the proximal surface of the radial carpal bone was the first area seen (Fig. 1).

Withdrawing of the arthroscope makes easier the visualization of the proximal surface of the intermediate carpal and the distal surface of the radius in its middle portion could be easily seen. The lateral joint angle could be seen from this portal with some difficulty, but it was better examined from the medial portal.

### Arthroscopic examination of the radiocarpal joint from the medial portal

**Fig. 2:**
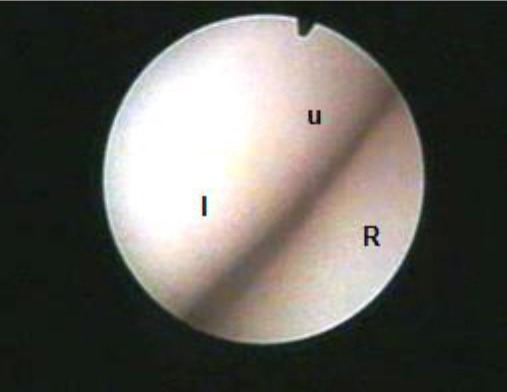
Arthroscopic view of the radiocarpal joint showing the lateral joint angle. (R): Radius; (U): Ulnar carpal bone; (I): Intermediate carpal bone.

Arthroscopic examination of the radiocarpal joint from the medial portal revealed that the first area seen was the lateral angle formed by the proximal articular surface of the ulnar carpal bone, the lateral aspect of the intermediate carpal bone and the distal articular surface of the radius (Fig. 2).

**Fig. 3:**
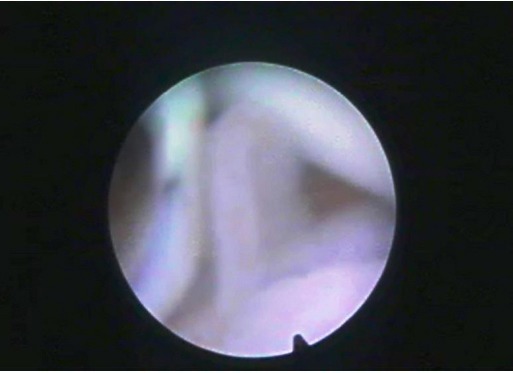
Arthroscopic view of the radiocarpal joint showing polyp like synovial villi.

The joint space, synovial membrane and villi could be easily examined. The shape of the villi varied from polyp like (Fig. 3), slender, stunted to finger like structures.

### Arthroscopic examination of group I

**Fig. 4:**
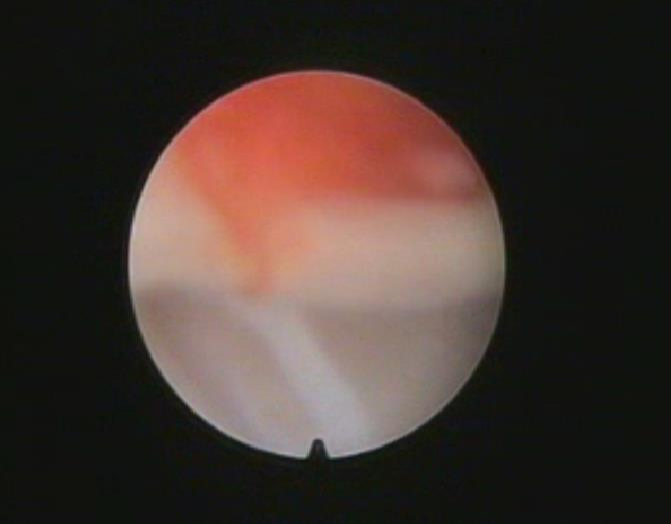
Arthroscopic view of the radiocarpal joint of a donkey from group I showing congestion of the synovial villi.

Arthroscopic examination of infected group I revealed that the cartilage remained clear and did not change. Mild degree of synovitis which was characterized by hyperemia and petechiation of the villi (Fig. 4) with slight degree of congestion and hypertrophied.

### Arthroscopic examination of group II

**Fig. 5:**
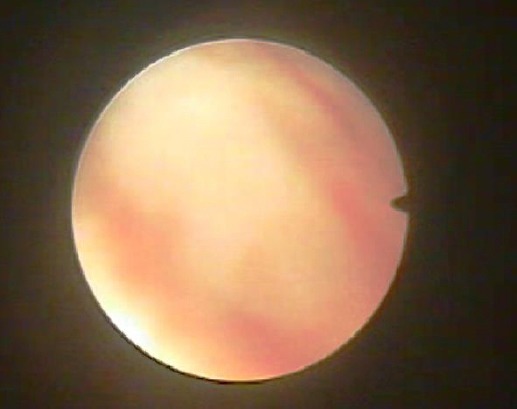
Arthroscopic view of the radiocarpal joint of a donkey from group II showing pannus

Arthroscopic examination of infected group II revealed the presence of a severe degree of synovitis which was characterized by hyperemia and petechiation of the villi. Severe degree of congestion and hypertrophied villi were present. Pannus (intra synovial fibrinocellular conglomerate) was present at this stage in addition to the beginning of articular cartilage erosion (Fig. 5).

### Arthroscopic examination of group III

**Fig. 6:**
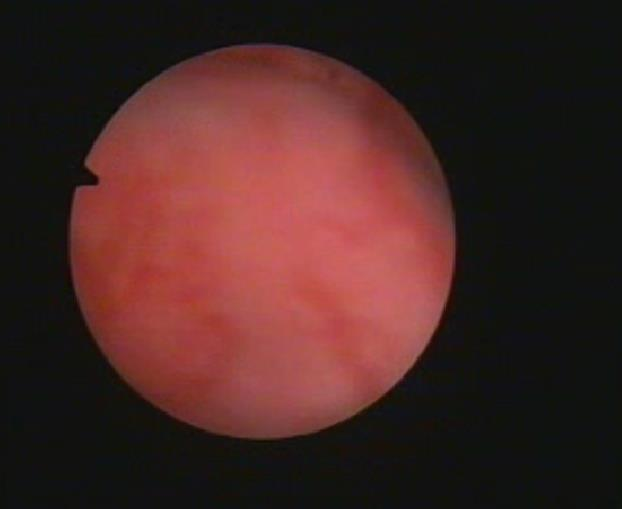
Arthroscopic view of the radiocarpal joint of a donkey from group III showing more prominent erosion of the articular cartilage.

Arthroscopic examination of infected group III revealed the presence of a more severe degree of synovitis characterized by hyperemia and petechiation of the villi. Severe degree of congestion which appear as patches, hypertrophied villi and more prominent erosion of articular cartilage was present as seen in Fig. 6.

## Discussion

In the present study, *Staph. aureus* was used in the induction of septic carpitis in donkeys because the most commonly isolated microorganisms from clinical cases was this microorganism. This study was performed in donkeys to examine the severity of every stage of septic carpitis and furthermore, use it for the clinical trials. These findings were in agreement with that reported by El-Maghraby and Al-Bawa’neh (2002).

The obtained results revealed that, the arthroscopic examination of the all aspects of the radiocarpal joint was accomplished through medial and lateral portals. This finding were in agreement with Magda, *et al*. (2005). However, Martin and McIlwraith (1985), McIlwraith (1990) and McIlwraith *et al*. (2005) recorded that adequate arthroscopic examination of the radiocarpal joint was possible through a single arthroscopic portal.

Using two separate portals, in the present study improved visualization of all joint structures by increasing the freedom of movement of arthroscope inside the joint cavity, reduction of soft tissue tension around the arthroscope and its tendency to slip out of the joint.

The lateral portal of the radiocarpal joint was positioned halfway between the extensor carpi radialis tendon and common digital extensor tendon at the level of the radiocarpal joint. The medial portal was located medial to the extensor carpi radialis tendon at the level of the radiocarpal joint. This finding were in agreement with Magda *et al*. (2005).

The same portals also were described by Martin and McIlwraith (1985), Hurtig and Fertz (1986), McIlwraith *et al*. (1987), McIlwraith (1990) and McIlwraith *et al*. (2005) in horses. Arthroscopic examination of the radiocarpal joint was best achieved with the joint flexed at 20 degrees. The same finding was recorded by Magda *et al*. (2005) in donkeys and also by Martin and McIlwraith (1985) and McIlwraith *et al*. (2005) in horses. Arthroscopic examination of the joint flexed at 20 degree reduces the tension of the synovial membrane on the dorsal aspects of the articular surfaces of the radial carpal bone, intermediate carpal bone and the radius.

Arthroscopic examination in cases of septic arthritis revealed synovitis which was characterized by hyperemia and petechiation of the villi. This finding were in agreement with McIlwraith (1984) who illustrated that synovitis was characterized by hyperaemia, petechiation of the villi, development of small hyperaemic villi in abnormal locations and new forms of villi. With severe inflammation, fusion of villi and the presence of fibrinoid strands may be observed.

Arthroscopic examination of septic carpitis (group II) revealed the presence of a severe degree of synovitis with hyperemia and petechiation of the villi. Severe degree of congestion and hypertrophied villi were present. Pannus was present at this stage in addition to the presence of erosion of the articular cartilage. These findings were in agreement with McIlwraith (1984), Wright (2002) and AbdEl-Glil (2012).

Arthroscopic examination of septic carpitis (group III) revealed presence of more severe degree of synovitis characterized by hyperemia and petechiation of the villi. Severe degree of congestion which appear as patches, hypertrophied villi and erosion were also present. These findings were in agreement with McIlwraith (1984) and AbdEl-Glil (2012).

In conclusion, arthroscopy provides advantages of diagnosis and management of infected joints to choose the suitable method of treatment.
